# Rethinking the Routine: Are Repeat Blood Cultures Necessary After Completion of Infective Endocarditis Treatment?

**DOI:** 10.1093/cid/ciaf617

**Published:** 2025-11-12

**Authors:** Jean Regina, Nicoleta Ianculescu, George Tzimas, Pierre Monney, Lars Niclauss, Matthias Kirsch, Benoit Guery, Matthaios Papadimitriou-Olivgeris

**Affiliations:** Department of Internal Medicine, Lausanne University Hospital and University of Lausanne, Lausanne, Switzerland; Department of Cardiology, Lausanne University Hospital and University of Lausanne, Lausanne, Switzerland; Department of Cardiology, Lausanne University Hospital and University of Lausanne, Lausanne, Switzerland; Department of Cardiology, Lausanne University Hospital and University of Lausanne, Lausanne, Switzerland; Department of Cardiac Surgery, Lausanne University Hospital and University of Lausanne, Lausanne, Switzerland; Department of Cardiac Surgery, Lausanne University Hospital and University of Lausanne, Lausanne, Switzerland; Infectious Diseases Service, Lausanne University Hospital and University of Lausanne, Lausanne, Switzerland; Infectious Diseases Service, Lausanne University Hospital and University of Lausanne, Lausanne, Switzerland; Infectious Diseases Service, Hospital of Valais and Institut Central des Hôpitaux, Sion, Switzerland

**Keywords:** infective endocarditis, Duke criteria, blood cultures, recurrence, bacteremia

## Abstract

Among 598 infective endocarditis (IE) episodes, follow-up blood cultures within 14 days of antimicrobial treatment completion were performed in 135 (23%) cases and detected only 2 (1.5%) recurrences. This strategy failed to identify 8 (6%) additional IE recurrences diagnosed between days 15 and 120, underscoring its limited utility.

**(See the Editorial Commentary by DeSimone and Baddour on pages e475–6.)**

Infective endocarditis (IE) is associated with high morbidity and mortality, with recurrence being one of its most severe complications. Recurrence occurs in approximately 2%–9% of cases and includes both relapse (due to the same pathogen) and reinfection (caused by a different pathogen) [1–8]. Relapse typically occurs within 6 months of the initial episode and is often related to insufficient treatment or persistent intracardiac infection, with *Staphylococcus aureus* and enterococcal IE being associated with higher relapse rates [1–7].

Both the European Society of Cardiology (ESC) and the American Heart Association (AHA) guidelines recommend performing blood cultures in any patient with symptoms suggestive of IE recurrence [2, 3]. However, they differ regarding asymptomatic patients: the ESC guidelines encourage routine blood cultures within the first week after completing antimicrobial therapy, even in the absence of symptoms, while the AHA guidelines recommend against routine blood cultures in asymptomatic patients, citing the low likelihood of detecting relapse [4, 5].

To date, no study has assessed whether clinicians perform repeat blood cultures after completion of IE treatment in clinical practice, or whether such cultures identify relapse in asymptomatic patients. The aim of this study was therefore to evaluate the prevalence of repeat blood cultures following IE treatment and to determine their diagnostic yield for detecting IE relapse.

## MATERIALS AND METHODS

This study was conducted at Lausanne University Hospital (CHUV), Switzerland, from January 2014 to June 2024. It included a retrospective cohort from 2014 to 2017 and a prospective cohort from 2018 to 2024. The study was approved by the Ethics Committee of the Canton of Vaud (CER-VD 2017-02137).

We included adults (aged ≥18 years) with IE and no recorded objection to data use (retrospective cohort) or who had provided written informed consent (prospective cohort). Exclusion criteria were as follows: IE caused by pathogens that do not grow in blood cultures or of unknown microbial etiology; patients on long-term suppressive antibiotic therapy; and patients who died during antimicrobial treatment or within 14 days after treatment completion, if no follow-up blood cultures were performed.

Demographic, clinical, imaging, surgical, and microbiological data were manually extracted from electronic health records and independently verified by an infectious diseases specialist (M. P.-O.). Both initial and recurrent IE episodes were diagnosed by the institution's Endocarditis Team based on the 2023 International Society of Cardiovascular Infectious Diseases Duke criteria [9]. At our institution, all IE episodes are followed by an infectious diseases consultant. The decision to perform follow-up blood cultures after completion of antimicrobial treatment, typically between 2 and 14 days, was left to the discretion of the treating physician. Early follow-up blood cultures were defined as those drawn within 14 days of treatment completion. Infective endocarditis recurrence within 120 days of treatment completion was recorded. Recurrent episodes caused by the same pathogen were classified as relapse, while those caused by a different pathogen, or with no pathogen identified, were classified as reinfection [6]. For all recurrent episodes (relapse or reinfection), the date of symptom onset was also collected.

SPSS version 26.0 (SPSS, Chicago, IL, USA) software and R software version 4.4.1 (R Core Team, Vienna, Austria) were used for data analysis. Categorical variables were analyzed using the χ² or Fisher exact test and continuous variables with Mann–Whitney *U* test. All statistic tests were 2-tailed, and *P* < .05 was considered statistically significant.

## RESULTS

**Figure 1.**
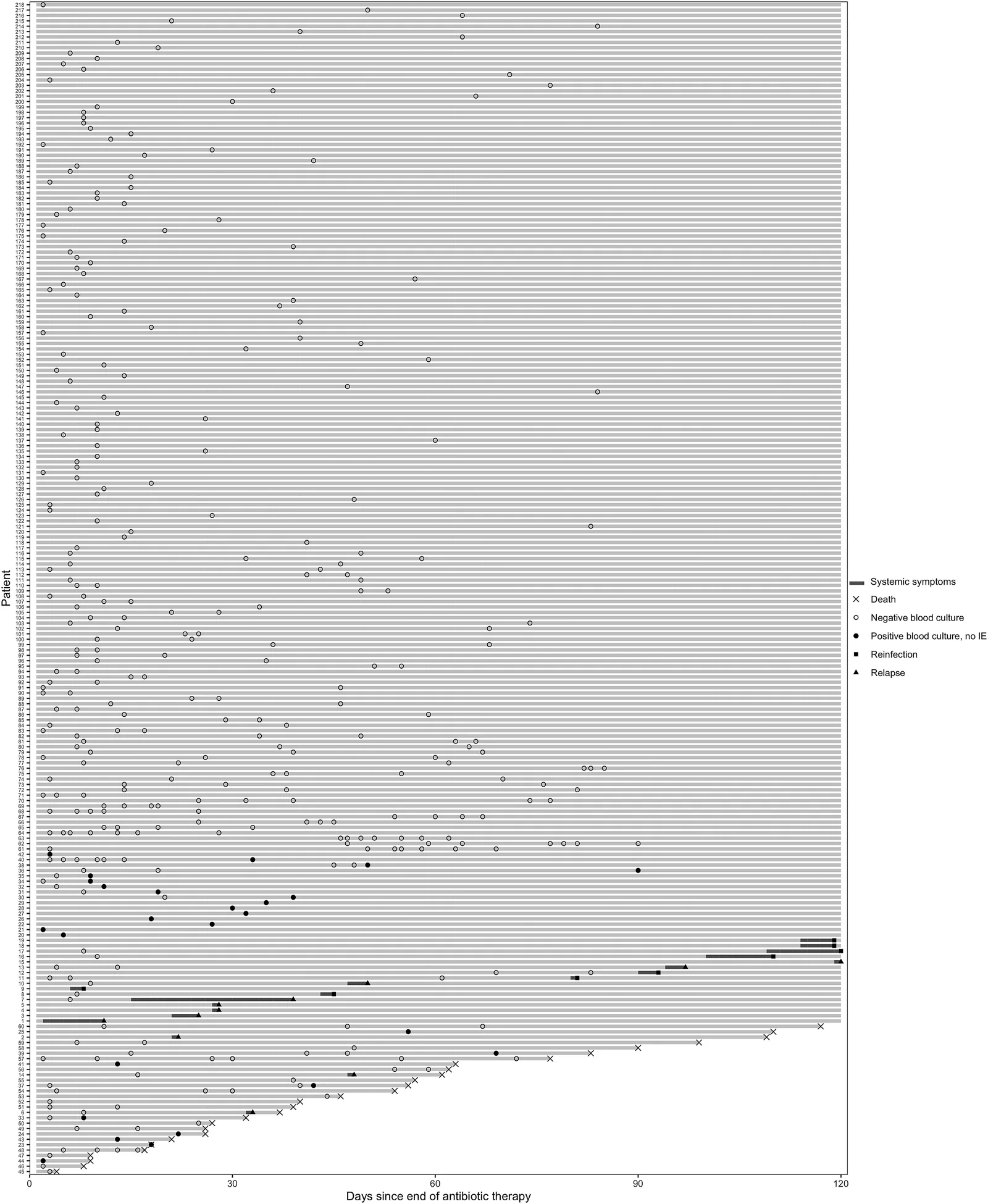
Follow-up blood cultures and recurrences (relapses or reinfections) of infective endocarditis after the completion of antimicrobial treatment.

Among 794 IE episodes, 598 were included (Supplementary Figure 1). Follow-up blood cultures within 90 days after completion of antimicrobial therapy were performed in 215 episodes (36%), with a median interval of 10 days (interquartile range 6–18 days) to the first blood culture (Figure 1).

Recurrence of IE within 120 days of treatment completion occurred in 19 episodes (3%): 11 were relapses caused by the same pathogen as the initial episode, and 8 were reinfections caused by different pathogens (Supplementary Table 1).

Early follow-up blood cultures, defined as those obtained within 14 days after treatment completion, were performed in 135 episodes (23%) and were positive in 12 episodes (9%). Of these, 10 were bacteremias caused by a different pathogen than in the initial episode, and IE was ruled out after multimodality imaging and evaluation by the Endocarditis Team, 1 episode was a relapse of IE caused by the same pathogen as the initial episode, and 1 episode was a reinfection by a different pathogen; both patients with recurrent IE (relapse or reinfection) were symptomatic at the time of blood culture collection. The remaining 17 episodes of recurrence (10 relapses and 7 reinfections) were diagnosed between 15 and 120 days after treatment completion. Among these, 8 patients (4 relapses and 4 reinfections) had early follow-up blood cultures that were negative.

Supplementary Table 2 summarizes the characteristics of episodes with and without early follow-up blood cultures. These cultures were more commonly performed in patients with a higher Charlson Comorbidity Index, enterococcal IE, and in those not undergoing valve surgery and/or cardiac implantable electronic device extraction.

## DISCUSSION

In our cohort, follow-up blood cultures were obtained within the first 2 weeks after treatment completion in 23% of IE episodes, reflecting partial adherence to the 2023 ESC guidelines, which recommend obtaining blood cultures in all patients during the immediate posttreatment period, even if asymptomatic [4]. The diagnostic yield of these early follow-up cultures was low, with only 2 (1.5%) recurrences identified through this approach, both in patients who were already symptomatic at the time of blood culture collection.

In addition to the low diagnostic yield, drawing blood cultures is time-consuming for clinical and laboratory staff, and they are susceptible to contamination by skin flora, with reported contamination rates ranging from 1% to 2% [10]. While some experts have suggested that blood culture collection may be of limited value when the likelihood of bacteremia is below 5% [11], this threshold is arbitrary and may not be applicable to patients convalescing from IE. In this population, any episode of bacteremia raises immediate concern for relapse or reinfection and often prompts repeat imaging, specialist evaluation, initiation of antimicrobial treatment, and consideration of surgical intervention. However, in our cohort, the probability of detecting IE recurrence with early follow-up blood cultures was 1%. Furthermore, this strategy failed to identify 8 (6%) additional IE recurrences diagnosed between days 15 and 120 after antimicrobial treatment completion. These findings therefore support the AHA's recommendation against routine follow-up blood cultures in asymptomatic patients, given the low likelihood of yielding meaningful results [3]. Nevertheless, as emphasized by both ESC and AHA guidelines, patients should be educated on the symptoms and signs of IE recurrence and instructed to seek immediate medical attention should these occur. In such instances, prompt blood culture collection is warranted, particularly if empiric antimicrobial therapy is being considered [4, 5].

The overall recurrence rate within 120 days after treatment completion was 3%, which is consistent with previous studies reporting recurrence rates between 2% and 9% [1–8]. Relapses occurred in 3% of *S. aureus* IE episodes, consistent with previously reported estimates, and in 2% of enterococcal IE episodes, lower than previously reported [7, 12]. This may be partly explained by our limited follow-up window of 120 days, as relapses of enterococcal IE—caused by low-virulence pathogens—may occur later [7, 12]. Despite this, infectious diseases specialist in our institution were more likely to perform early follow-up blood cultures in patients with enterococcal IE than in those with other causative pathogens.

This study has several limitations. First, it was a single-center study, and part of the cohort was included retrospectively. However, to the best of our knowledge, this is the first study to specifically address the knowledge gap on follow-up blood cultures after IE treatment completion. Second, follow-up blood culture collection was not systematic but rather reflected individual clinician judgment and patient characteristics. Third, all patients with IE were managed by infectious diseases consultants and evaluated by a dedicated Endocarditis Team, with access to advanced imaging techniques, which may limit the generalizability of our findings to other settings [13]. This multimodality imaging approach, combined with subsequent evaluation by the Endocarditis Team, permitted the diagnosis of IE recurrences and the exclusion of IE in several episodes of bacteremia. Fourth, we only had access to follow-up blood cultures performed at Lausanne University Hospital and may have missed cultures obtained at outside facilities. However, given our structured follow-up procedures, we are confident that no recurrence of IE was missed. Lastly, we did not systematically record whether patients were symptomatic at the time follow-up blood cultures were drawn. This information was only available for patients with IE recurrence. Nevertheless, given institutional practice, where infectious diseases consultants typically request follow-up blood cultures between days 2 and 14 posttreatment, we assume that most of these cultures were drawn in asymptomatic patients.

In summary, our findings suggest that routine early follow-up blood cultures in asymptomatic patients after completion of antimicrobial therapy for IE have limited diagnostic yield and align with the AHA's recommendation against their systematic use in this group. Future studies should aim to identify which subgroups of asymptomatic patients may benefit from follow-up blood cultures and to define the optimal timing of their collection after completion of antimicrobial therapy, with the goal of maximizing relapse detection while minimizing unnecessary investigations.

## Supplementary Material

ciaf617_Supplementary_Data

## Supplementary Data

Supplementary materials are available at *Clinical Infectious Diseases* online. Consisting of data provided by the authors to benefit the reader, the posted materials are not copyedited and are the sole responsibility of the authors, so questions or comments should be addressed to the corresponding author.
