# Supplementary material for: Rethinking the Routine: Are Repeat Blood Cultures Necessary After Completion of Infective Endocarditis Treatment?
Source: Clin Infect Dis. 2025 Nov 12;82(3):e471–4. doi: 10.1093/cid/ciaf617 (PMC13016772; doi:10.1093/cid/ciaf617)
Supplement: ciaf617_Supplementary_Data [file ciaf617_supplementary_data.pdf]

**Supplementary Figure 1.**

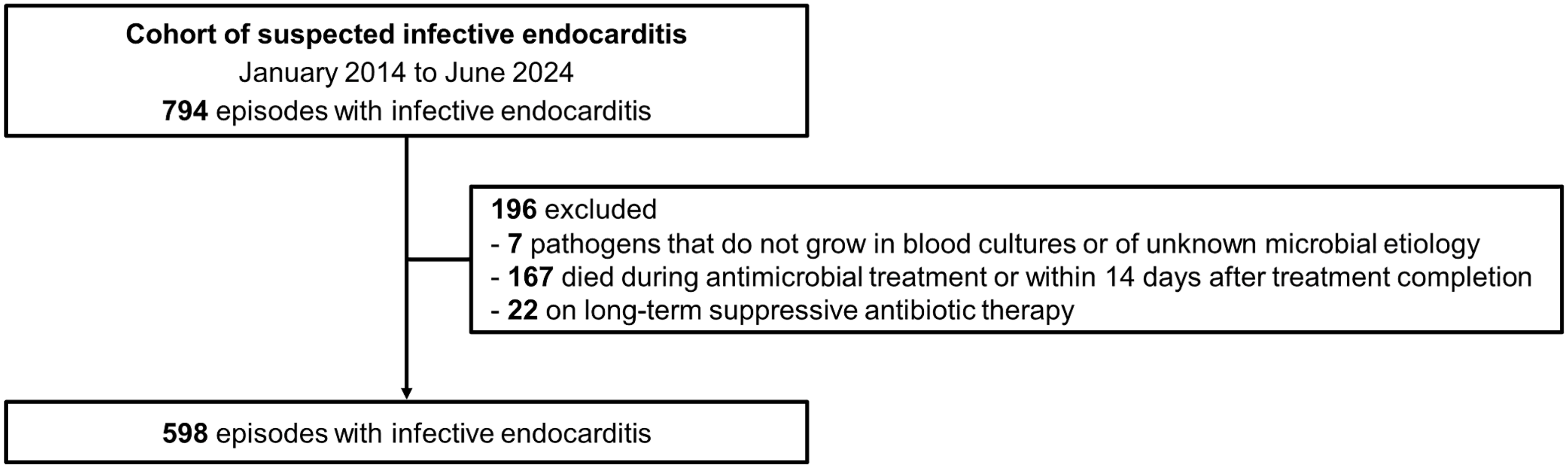

**Supplementary Table 1.** Characteristics of episodes with positive repeat blood cultures within 90 days and/or recurrence of infective endocarditis within 120 days following antimicrobial treatment completion

| Episode | Initial IE episode | | Blood cultures after antimicrobial treatment completion | | | | New IE episode | | |
|---|---|---|---|---|---|---|---|---|---|
| | Pathogen | Type | Days | Days of positive | Pathogen | Type | Days of symptom onset | Pathogen | Type |
| 1 | *P. aeruginosa* | PVE | 11 | 11 | *P. aeruginosa* | Relapse of PVE | 2 | *P. aeruginosa* | Relapse of PVE |
| 2 | *S. epidermidis* | NVE | 22 | 22 | *S. epidermidis* | Relapse of NVE | 21 | *S. epidermidis* | Relapse of NVE |
| 3 | *S. aureus* | PVE | 25 | 25 | *S. aureus* | Relapse of PVE | 21 | *S. aureus* | Relapse of PVE |
| 4 | *E. faecalis* | NVE | 28 | 28 | *E. faecalis* | Relapse of NVE | 27 | *E. faecalis* | Relapse of PVE |
| 5 | *S. aureus* | NVE | 28 | 28 | *S. aureus* | Relapse of NVE | 27 | *S. aureus* | Relapse of NVE |
| 6 | *S. aureus* | NVE | 8, 33 | 33 | *S. aureus* | Relapse of NVE | 32 | *S. aureus* | Relapse of NVE |
| 7 | *S. mitis* | NVE | 6, 39 | 39 | *S. mitis* | Relapse of NVE | 15 | *S. mitis* | Relapse of NVE |
| 8 | *S. aureus* | PVE | 7, 45 | | | | 43 | No identification | Reinfection PVE |
| 9 | *S. aureus* | NVE | 8 | 8 | *S. mitis* | Reinfection PVE | 6 | *S. mitis* | Reinfection PVE |
| 10 | *S. aureus* | NVE | 9, 50 | 50 | *S. aureus* | Relapse of NVE | 47 | *S. aureus* | Relapse of PVE |
| 11 | *S. aureus* | PVE | 3, 6, 61, 81 | | | | 80 | No identification | Reinfection PVE |
| 12 | *E. cloacae* | PVE | 69, 83 | | | | 91 | *S. cerevisiae* | Reinfection PVE |
| 13 | *S. aureus* | NVE | 4, 13 | | | | 94 | *S. aureus* | Relapse of PVE |
| 14 | *E. faecalis* | PVE | 16, 48 | 48 | *E. faecalis* | Relapse of PVE | 47 | *E. faecalis* | Relapse of PVE |
| 15 | *S. aureus* | NVE, CIED-IE | - | | | | 119 | *S. aureus* | Relapse NVE |

| | | | | | | | | | |
|---|---|---|---|---|---|---|---|---|---|
| 16 | *E. faecalis* | PVE | 10 | | | | 100 | *S. gallolyticus* | Reinfection PVE |
| 17 | *S. aureus* | NVE | 8 | | | | 109 | *S. dysgalactiae* | Reinfection PVE |
| 18 | *S. aureus* | NVE | - | | | | 114 | No identification | Reinfection NVE |
| 19 | *S. mitis* | PVE | - | | | | 119 | No identification | Reinfection PVE |
| 20 | *E. faecalis* | NVE | 5 | 5 | *K. aerogenes* | No IE | | | |
| 21 | *S. sanguinis* | PVE | 2 | 2 | *K. pneumoniae* | No IE | | | |
| 22 | *E. faecalis* | NVE | 27 | 27 | *E. coli* | No IE | | | |
| 23 | *S. aureus* | CIED-IE | 18 | 18 | *E. cloacae* | No IE | | | |
| 24 | *S. aureus* | NVE | 22 | 22 | *E. coli* | No IE | | | |
| 25 | *S. aureus* | NVE | 56 | 56 | *K. aerogenes* | No IE | | | |
| 26 | *S. aureus* | NVE | 18 | 18 | *E. coli* | No IE | | | |
| 27 | *S. mitis* | NVE | 32 | 32 | *K. pneumoniae* | No IE | | | |
| 28 | *C. hominis* | PVE | 30 | 30 | *S. mitis* | No IE | | | |
| 29 | *S. aureus* | NVE | 35 | 35 | *E. coli* | No IE | | | |
| 30 | *S. mitis* | NVE, PVE | 20, 39 | 39 | *E. faecalis* | No IE | | | |
| 31 | *E. faecalis* | PVE | 8, 19 | 19 | *E. cloacae* | No IE | | | |
| 32 | *S. mutans* | NVE | 4, 11 | 11 | *E. faecalis* | No IE | | | |
| 33 | *S. gallolyticus* | NVE | 3, 8 | 8 | *E. faecium* | No IE | | | |
| 34 | *S. epidermidis* | PVE | 2, 9 | 9 | *E. faecium* | No IE | | | |
| 35 | *E. faecalis* | PVE | 4, 9 | 9 | *E. coli* | No IE | | | |

| | | | | | | |
|---|---|---|---|---|---|---|
| 36 | *E. faecalis* | PVE | 8, 19, 90 | 90 | *S. anginosus* | No IE |
| 37 | *E. faecalis* | PVE | 3, 40, 42 | 42 | *E. faecium* | No IE |
| 38 | *S. anginosus* | NVE | 45, 48, 50 | 50 | *E. faecium* | No IE |
| 39 | *S. aureus* | NVE | 15, 41, 47, 69 | 69 | *E. coli* | No IE |
| 40 | *S. epidermidis* | PVE | 3, 5, 7, 10, 11, 14, 33 | 33 | *E. faecalis* | No IE |
| 41 | *E. faecium* | PVE | 13 | 13 | *P. aeruginosa* | No IE |
| 42 | *S. aureus* | NVE | 3 | 3 | *E. coli* | No IE |
| 43 | *E. faecalis* | CIED-IE | 13 | 13 | *S. epidermidis* | No IE |
| 44 | *S. gallolyticus* | NVE | 2 | 2 | *S. mitis* | No IE |

IE: infective endocarditis, NVE: native valve endocarditis, PVE: prosthetic valve endocarditis, CIED-IE: cardiac implantable electronic device-related infective endocarditis

Full names of pathogens mentioned in this table (alphabetical order): *Cardiobacterium hominis*, *Enterobacter cloacae*, *Enterococcus faecalis*, *Enterococcus faecium*, *Escherichia coli*, *Klebsiella aerogenes*, *Klebsiella pneumoniae*, *Pseudomonas aeruginosa*, *Saccharomyces cerevisiae*, *Staphylococcus aureus*, *Staphylococcus epidermidis*, *Streptococcus anginosus*, *Streptococcus dysgalactiae*, *Streptococcus gallolyticus*, *Streptococcus mitis*, *Streptococcus mutans*, *Streptococcus sanguinis*.

**Supplementary Table 2.** Comparison of episodes with or without follow-up blood cultures performed within 14 days after antimicrobial treatment completion

| | **No blood cultures (n=463)** | **Blood cultures (n=135)** | ***P*** |
|---|---|---|---|
| Demographics | | | |
| Male sex, n (%) | 342 (74) | 103 (76) | 0.654 |
| Age (years), median (IQR) | 67 (52-76) | 68 (56-78) | 0.202 |
| Age >60 years, n (%) | 290 (63) | 90 (67) | 0.417 |
| Co-morbidities | | | |
| Malignancy (solid organ or hematologic), n (%) | 47 (10) | 23 (17) | 0.033 |
| Immunosuppression, n (%)[a] | 20 (4) | 13 (10) | 0.030 |
| Diabetes mellitus, n (%) | 98 (21) | 32 (24) | 0.554 |
| Chronic kidney disease (moderate or severe), n (%) | 72 (16) | 31 (23) | 0.052 |
| Obesity (body mass index ≥30 kg/m$^2$), n (%) | 82 (18) | 32 (24) | 0.135 |
| Chronic obstructive pulmonary disease, n (%) | 46 (10) | 24 (18) | 0.021 |
| Congestive heart failure, n (%) | 56 (12) | 13 (10) | 0.540 |
| Cirrhosis, n (%) | 27 (6) | 9 (7) | 0.684 |
| Intravenous drug use, n (%) | 46 (10) | 8 (6) | 0.175 |
| Charlson Comorbidity Index (points), median (IQR) | 4 (1-6) | 5 (2-7) | 0.038 |

| | | | |
|---|---|---|---|
| Charlson Comorbidity Index >4 points, n (%) | 187 (40) | 70 (52) | 0.023 |
| Setting of infection onset | | | 0.232 |
| Community-acquired, n (%) | 348 (75) | 94 (70) | |
| Healthcare-associated, n (%) | 59 (13) | 25 (19) | |
| Nosocomial, n (%) | 56 (12) | 16 (12) | |
| Microbiological data | | | |
| *Staphylococcus aureus*, n (%) | 167 (36) | 50 (37) | 0.839 |
| Coagulase negative staphylococci, n (%) | 25 (5) | 12 (9) | 0.155 |
| Streptococci, n (%) | 129 (28) | 29 (22) | 0.150 |
| Enterococci, n (%) | 61 (13) | 32 (24) | 0.004 |
| Other Gram-positive, n (%) | 10 (2) | 3 (2) | 1.000 |
| HACEK, n (%) | 17 (4) | 1 (0.7) | 0.090 |
| Other Gram-negative, n (%) | 16 (4) | 6 (4) | 0.605 |
| Resistant bacteria, n (%)[b] | 34 (7) | 12 (9) | 0.582 |
| Persistent bacteremia (≥48h), n (%) | 128 (8) | 4- (30) | 0.664 |
| No pathogen identification, n (%)[c] | 44 (10) | 8 (6) | 0.227 |
| Infection data | | | |
| Fever, n (%) | 369 (80) | 115 (85) | 0.172 |
| Sepsis, n (%) | 196 (42) | 48 (36) | 0.165 |

| | | | |
|---|---|---|---|
| Embolic events, n (%) | 231 (50) | 65 (48) | 0.769 |
| Immunological phenomena, n (%) | 34 (7) | 7 (5) | 0.444 |
| Bone and joint infection, n (%) | 110 (24) | 25 (19) | 0.242 |
| Site of infective endocarditis | | | |
| Native valve, n (%) | 308 (67) | 85 (63) | 0.471 |
| Prosthetic valve, n (%) | 108 (23) | 39 (29) | 0.211 |
| Cardiac implantable electronic device lead, n (%) | 64 (14) | 16 (12) | 0.667 |
| Types of cardiac lesion | | | |
| Vegetation, n (%) | 292 (63) | 83 (762) | 0.762 |
| Vegetation ≥10mm, n (%) | 221 (48) | 55 (41) | 0.170 |
| Abscess, n (%) | 90 (19) | 23 (17) | 0.617 |
| Other lesions, n (%)[d] | 76 (16) | 22 (16) | 1.000 |
| Management of initial episode | | | |
| Valve surgery, and/or CIED-extraction, n (%) | 241 (52) | 54 (40) | 0.015 |
| Infective endocarditis within 120 days, n (%) | | | 0.004 |
| No/died within 120 days, n (%) | 35 (8) | 15 (11) | |
| No/survived at least 120 days, n (%) | 419 (91) | 111 (83) | |
| Yes/same pathogen as initial episode, n (%) | 6 (1) | 5 (4) | |
| Yes/different pathogen than initial episode, n (%) | 3 (0.6) | 5 (4) | |

CIED: cardiac implantable electronic device; IQR, Interquartile range

[a]ongoing immunosuppressive treatment at bacteremia onset, intravenous chemotherapy in the 30 days prior to bacteremia onset, AIDS, neutropenia and asplenia.

[b]methicillin resistant staphylococci, penicillin resistant streptococci, or amoxicillin resistant enterococci

[c]blood culture negative infective endocarditis due to antimicrobial administration prior to blood culture collection

[d]perforation, dehiscence of prothesis, aneurysm, pseudoaneurysm, or fistula
